# Pharmacological induction of cell surface GRP78 contributes to apoptosis in triple negative breast cancer cells

**DOI:** 10.18632/oncotarget.2576

**Published:** 2014-10-24

**Authors:** Annat Raiter, Rinat Yerushalmi, Britta Hardy

**Affiliations:** ^1^ Felsenstein Medical Research Center, Tel Aviv University School of Medicine, Rabin Medical Center, Petach Tikva, 49100, Israel; ^2^ Oncology Institute, Rabin Medical Center, Petach Tikva, 49100, Israel

**Keywords:** triple negative breast cancer cells, drug induced cell surface GRP78, apoptosis

## Abstract

Breast cancer tumor with triple-negative receptors (estrogen, progesterone and Her 2, receptors) is the most aggressive and deadly subtype, with high rates of disease recurrence and poor survival.

Here, we show that induction in cell surface GRP78 by doxorubicin and tunicamycin was associated with CHOP/GADD153 upregulation and increase in apoptosis in triple negative breast cancer tumor cells.

GRP78 is a major regulator of the stress induced unfolded protein response pathway and CHOP/GADD153 is a pro-apoptotic transcription factor associated exclusively with stress induced apoptosis.

The blocking of cell surface GRP78 by anti-GRP78 antibody prevented apoptosis, suggesting that induction of cell surface GRP78 by doxorubicin and tunicamycin is required for apoptosis.

A better understanding of stress induction of apoptotic signaling in triple negative breast cancer cells may help to define new therapeutic strategies.

## INTRODUCTION

Breast cancer is the most commonly diagnosed cancer among women worldwide [1,2]. The phenotypic manifestations of the disease differ in patients with diverse cancer subtypes that exhibit varied responses to different treatment modalities [3]. Breast cancers are divided into subgroups and subtypes based on the expression of three cell surface receptors: estrogen receptor (ER), progesterone receptor (PR) and human epidermal growth factor receptor 2(HER2). Tumor response to most existing treatments and therapy strategy depends on the status of these three therapeutically relevant receptors [4,5]. The luminal subtype shows higher expression of ER and indicates a favorable prognosis. When the three receptors are absent (triple-negative) or at low levels as in the basal-like tumors, a poor prognosis is diagnosed [6]. The two most common treatments include anti-estrogens for ER tumors and the monoclonal antibody anti Her2 for HER2 tumors with or without chemotherapy. In fact, the only option to treat triple negative breast cancer (TNBC) is non-specific and highly toxic chemotherapy or radiation therapy [7, 8]. Numerous experimental approaches are being attempted to identify targets in TNBC, including PI3K inhibitors, MEK inhibitors, HSP-90 inhibitors, histone deacetylase inhibitors, PARP inhibitors and PD-1 inhibitors. However, TNBC is a complex disease that requires the combination of two or more targets with different signaling pathways for an optimal treatment approach [9].

Glucose-regulated protein 78 kDa (GRP78) is a member of the heat shock protein 70 family that functions as a chaperone for folding, maturation and transport of polypeptides and proteins in the endoplasmic reticulum [10–14].

GRP78 is also a key member of the unfolded protein response (UPR) that protects cells from undergoing apoptosis under physiological stress conditions [15,16]. As an adaptive response to endoplasmic reticulum stress, the UPR activates a set of pathways that result in the activation of inositol-requiring protein1 (IRE1), protein kinase RNA-like endoplasmic reticulum kinase (PERK) and activating transcription factor 6 (ATF6). Activtion of these pathways selectively suppress protein synthesis, promote the translation of specific proteins and regulate a wide variety of UPR target genes expression, including GRP78 and the major pro-apoptotic transcription factor CHOP (also called GADD153) [17–19].

The induction of GRP78 by stress leads to an increase in GRP78 in the endoplasmic reticulum compartment, as well as its re-localization to the cell surface of various types of cancer including breast cancer cells (Hs578T, MDA-MB-231, BrCa-MZ-01 and MCF-7) [12, 18].

Cell surface GRP78 expression in several tumors was correlated with a poor prognosis and resistance to chemotherapy agents [20–26]. Controversially, other studies associated increased GRP78 expression with the efficacy of breast cancer treatments. One study described GRP78 as a novel positive predictor for breast cancer sensitivity to doxorubicin/taxane-based adjuvant chemotherapy [27]. Another study reported that aberrant induction of GRP78 correlated with resistance to chemotherapeutic agents, such as 5-fluorouracil, doxorubicin and etoposide, preventing apoptosis by blocking procaspase-7 [28]. In this study we aim to elucidate the significance of cell surface GRP78 in tumor cell growth and apoptosis using doxorubicin and tunicamycin, drugs involved in UPR signaling pathway, to induce the increase in cell surface GRP78 levels in a TNBC cancer cell line.

## RESULTS

### GRP78 expression: Comparison between MDAMB468 (triple negative) and BT474 (PR, ER and HER2 positive) breast cancer cell lines

**Figure 1:**
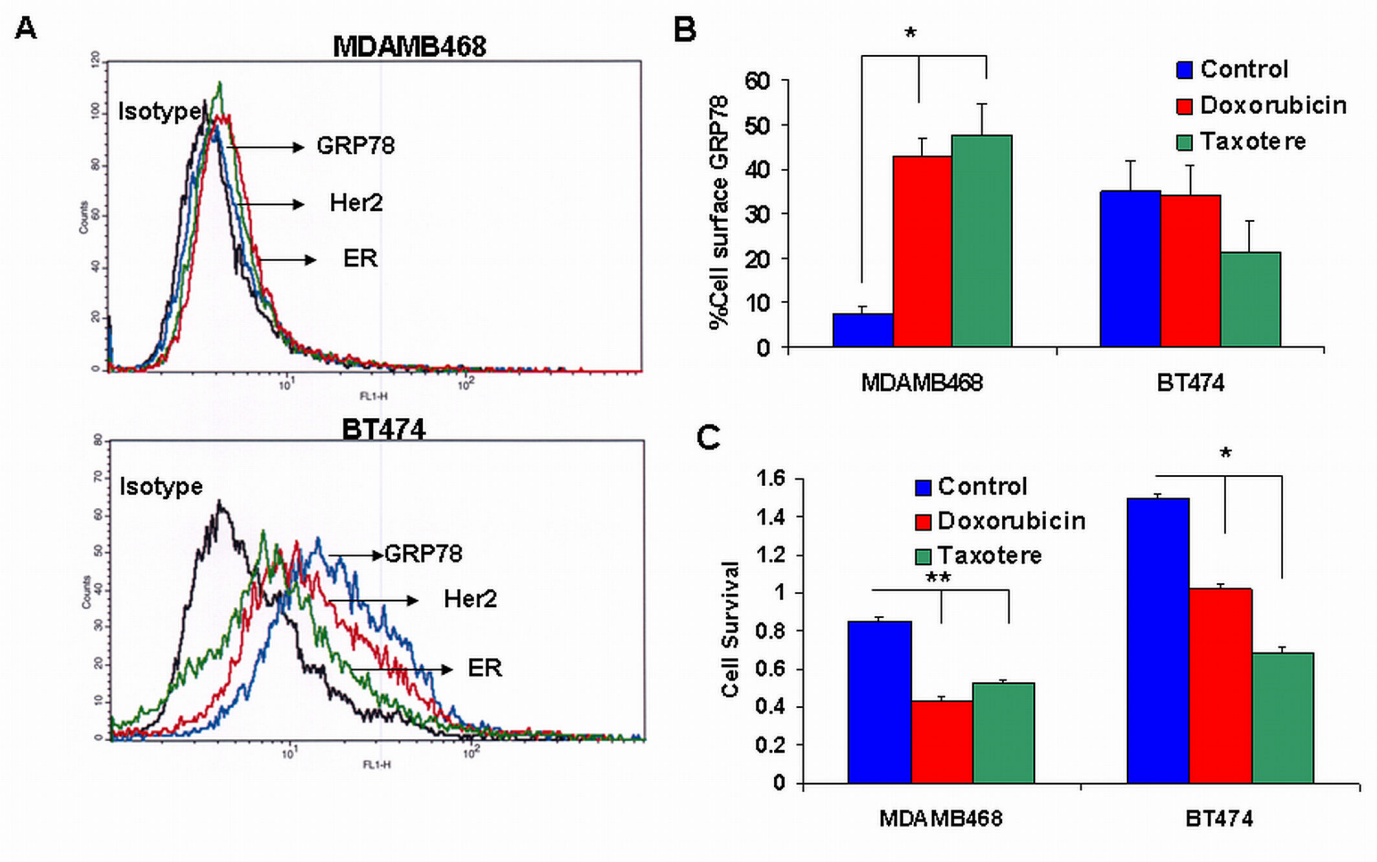
GRP78 expression: Comparison between MDAMB468 and BT474 breast cancer cell lines **(A)** Representative histograms by FACS analysis showing MDAMB468 cell line negative for cell surface Her2 (red line), ER (green line) and GRP78 (blue line) and BT474 positive for cell surface Her2, ER and GRP78. Isotype control is represented by a black line. **(B)** Effect of doxorubicin and taxotere on the percent of cell surface GRP78. Doxorubicin and taxotere significantly increased the percent of cell surface GRP78 in MDAMB468 triple negative cells (*p < 0.001) but not in BT474 cells. **(C)** The effect of the different drugs on cell survival. BT474 and MDAMB468 were demonstrated to be sensitive to doxorubicin. The effect of taxotere was greater in BT474 than in MDAMB468 cells. The addition of taxotere significantly decreased cell survival in BT474 cells (*p < 0.001) and but was less effective, though significant in MDAMB468 cells (**p < 0.05).

We compared two different human breast carcinoma cell lines: MDAMB468 negative basal carcinoma and BT474 luminal subtype cell line for the expression of cell surface GRP78. FACS analysis was performed with the specific antibodies to determine the presence of Her-2, ER and cell surface GRP78 on cells. Figure 1A shows that MDAMB468 cells are negative for all 3 receptors (GRP78 5.7 ± 2.2%). In contrast, BT474 was positive for cell surface GRP78 (58.5 ± 6.6%) correlating with HER2 and ER positive expression.

To evaluate the effect of drugs on cell surface GRP78 expression we incubated the cells with doxorubicin and taxotere. Surprisingly, we found that doxorubicin (0.1 μg/ml) and taxotere (5 μg/ml) significantly increased cell surface GRP78 expression on MDAMB468 (50.0 ± 7.7% and 55.3 ± 18.3% respectively; p < 0.001). GRP78 expression did not change in BT474 treated cells (Figure 1B). The effect of the different drugs on cell survival was determined by XTT proliferation. BT474 and MDAMB468 were demonstrated to be sensitive to doxorubicin. Taxotere had a greater effect on BT474 compared to MDAMB468. The addition of taxotere showed a 2.2-fold decrease in cell proliferation in BT474 cells ( *p* < 0.001), in contrast to a 1.6-fold decrease in MDAMB468 ( *p* < 0.05) (Figure 1C).

### Cell surface GRP78 on negative cell lines induced by doxorubicin and tunicamycin

**Figure 2:**
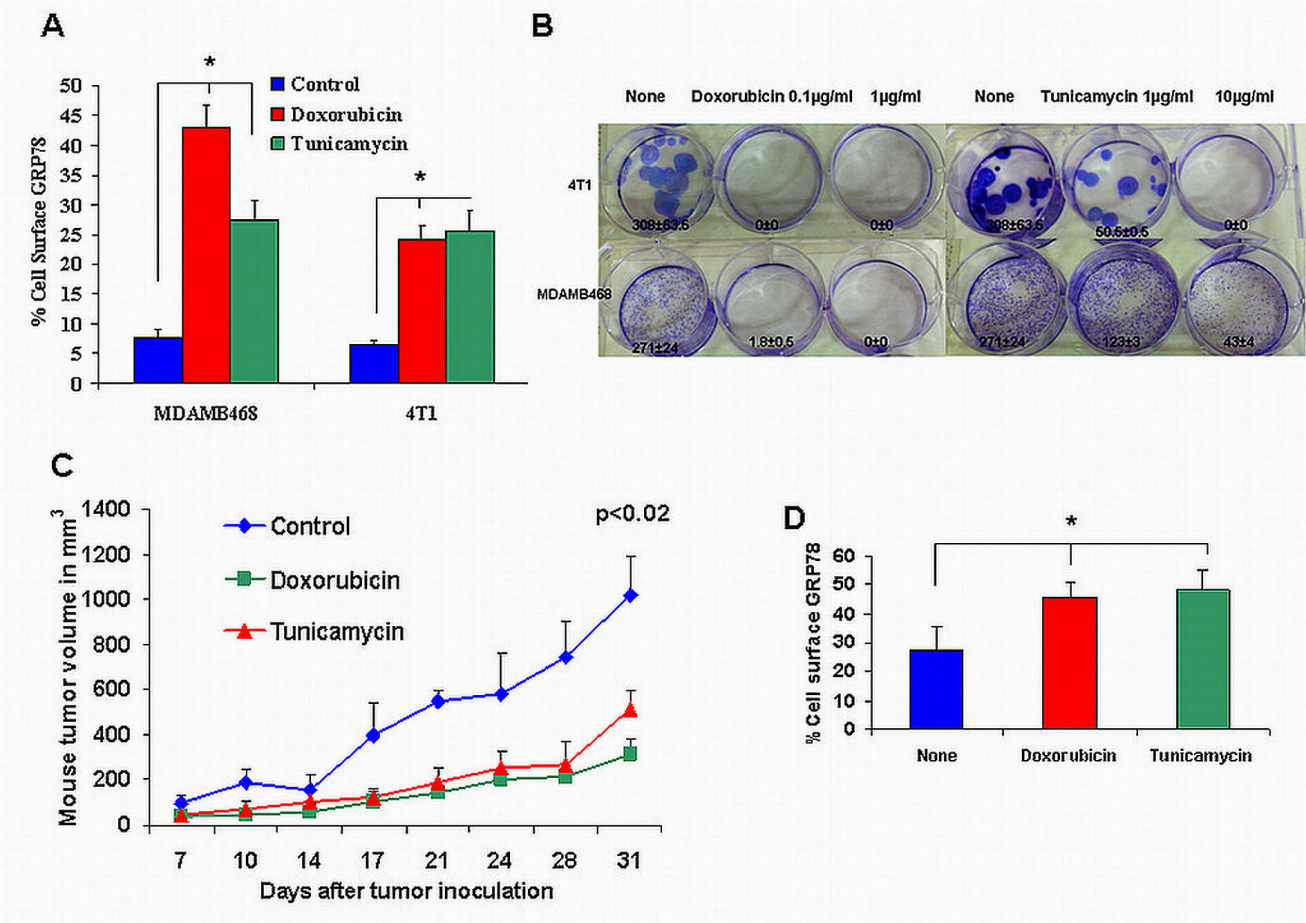
Tumorigenic effect of doxorubicin and tunicamycin on cell surface GRP78 negative cell lines **(A)** The 4T1 breast cancer mouse cell line expressed a low percent of cell surface GRP78 similar to MDAMB468. Doxorubicin and tunicamycin induced a significant increase in cell surface GRP78 (*p < 0.001). **(B)** Colony formation by MDAMB468 and 4T1 TNBC cells treated with doxorubicin and tunicamycin was inhibited significantly (*p < 0.001). **(C)** 10-week-old Balb/C nude mice were inoculated subcutaneously in the right flank with 1 × 10^6^ 4T1 cells in 100 μL PBS or with 4T1 pre-incubated with 0.1 μg/ml doxorubicin or with 10 μg/ml tunicamicin (10 mice per group). Mice from the same group uniformly developed relatively small tumors after doxorubicin or tunicamycin treatment compared to non treated mice cells (*p* < 0.02). **(D)** 4T1 cells extracted from mice xenografts, 31 days after tumor inoculation, showed significant increased cell surface GRP78 pre-incubated with doxorubicin (0.1 μg/ml) or tunicamycin (10 μg/ml) (*p < 0.004).

Since doxorubicin and tunicamycin were described to induce UPR signal transduction in which GRP78 plays a key role, we carried on our experiments using these drugs. We studied the induction of cell surface GRP78 expression on the negative mouse breast cancer cell line 4T1. The results obtained were similar to those of the human MDAMB468 cells. Figure 2A shows that a 6.4 ± 0.8 percent of 4T1 cells expressing cell surface GRP78 was raised by doxorubicin (0.1 μg/ml) to 28.2 ± 2.13% (*p* < 0.001). Similarly, tunicamycin increased cell surface GRP78 expression in both human MDAMB468 and mouse 4T1 cell lines to 27.4 ± 3.3% and 30.4 ± 3.45% respectively (*p* < 0.001).

### The effect of doxorubicin and tunicamycin on 4T1 cells tumorigenesis

Tumorigenesis was evaluated by in vitro colony formation and by in vivo tumor growth. Cells incubated with doxorubicin at 0.1 or 1 μg/ml completely restrained 4T1 colony formation. Tunicamycin at 1 μg/ml reduced colony formation in 4T1 cells by 6-fold (*p* < 0.001) and completely at 10 μg/ml (Figure 2B). Similar results were obtained with MDAMB468 cells incubated in the presence of 0.1 μg/ml doxorubicin and 10 μg/ml tunicamycin. Colony formation was reduced by 2.2-fold and 6.3-fold respectively.

For tumor growth, we monitored for 31 days the size of tumor nodules developed by 4T1 cells inoculated subcutaneously. Cells were incubated for 48 hs with 0.1 μg/ml doxorubicin and 10 μg/ml tunicamycin prior to inoculation in order to induce increased cell surface GRP78. Identical numbers of live cells were inoculated to mice in order to compare tumor growth in the 3 groups. Figure 2C shows a significant ( *p* < 0.02) decrease in tumor growth in doxorubicin (group 2) and tunicamycin (group 3) pretreated 4T1.

We evaluated the cell surface GRP78 on cells extracted from the tumor nodules 31 days after tumor inoculation. Cells showed a significant ( *p* < 0.004) increase from 27.4 ± 2.01% in control mice (group 1) to 45.7 ± 2.5% in pre-treated cells with doxorubicin and 48.3 ± 3.5% in cells pretreated with tunicamycin (Figure 2D).

### The correlation between cell surface GRP78 and cell apoptosis

The effect of doxorubicin and tunicamycin on 4T1 cell apoptosis was tested in cell cultures after GRP78 cell surface induction by the drugs and in the cells isolated from the tumors developed in the mice after 31 days.

**Figure 3:**
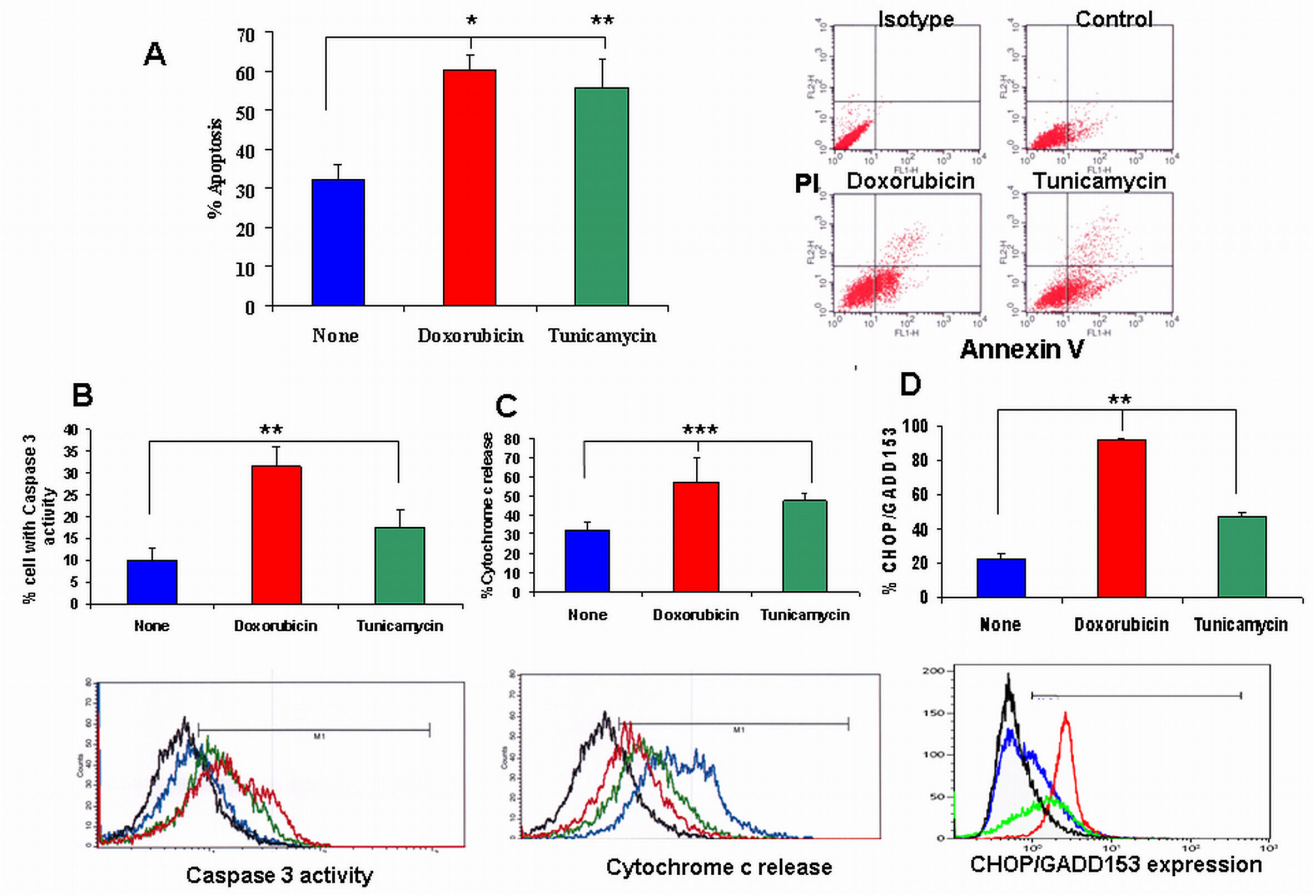
The effect of doxorubicin and tunicamycin on apoptosis **(A)** Determination of the effect of doxorubicin and tunicamycin on apoptosis by FACS analysis. Doxorubicin (0.1 μg/ml) significantly induced apoptosis in 4T1 cells (**p < 0.001). Tunicamycin induced apoptosis significantly at 10 μg/ml (*p < 0.05). On the upper right, representative FACS analysis of apoptosis by AnnexinV/PI of cells incubated with doxorubicin and tunicamycin compared to non-treated cells. **(B)** Caspase 3 activity was significantly increased by these drugs (**p < 0.001). **(C)** Cytochrome c release was increased significantly by these drugs (***p < 0.006). **(D)** CHOP/GADD153 expression was significantly increased by doxorubicin and tunicamycin (**p < 0.001). Representative FACS analysis of caspase 3 activity, cytochrome c detection and CHOP/GADD153 expression in 4T1 cells treated with doxorubicin and tunicamycin compared to non-treated cells. Black line: Isotype. Blue line: Non treated control cells. Red line: Doxorubicin treated cells. Green line: Tunicamycin treated cells.

In Figure 3A, we present FACS analysis of the apoptotic cells consequent to drug treatment. As can be seen, 0.1 μg/ml doxorubicin induced 60.3 ± 1.9 percent apoptosis in 4T1 cells ( *p* < 0.001). A higher dose of 1 μg/ml resulted in 71.2 ± 9.5% apoptotic cells ( *p* < 0.001). Similar results were obtained with MDAMB468 cells (data not shown). A significant induction of apoptosis was obtained by 10 μg/ml tunicamycin. Analysis of apoptosis revealed that 85% of the apoptotic cells that were incubated with 0.1 μg/ml doxorubicin and 10 μg/ml tunicamycin belong to early apoptosis (AnnexinV positive cells) and only 15% were necrotic (Annexin V/PI positive cells). Since doxorubicin at a higher dose, increased the percent of necrotic cells to 50%, we decided to use 0.1 μg/ml doxorubicin and 10 μg/ml tunicamycin to evaluate the tumor growth. We analyzed caspase 3 activity and cytochrome c release involved in apoptosis, in 4T1 tumor cells obtained from 31 days tumor nodules. As seen in Figure 3B, there was a significant increase in caspase 3 activity in cells pre-treated with doxorubicin (34.4 ± 4.7%; *p* < 0.001) and tunicamycin (17.4 ± 4.3; *p* < 0.05) in comparison to control cells (9.9 ± 2.7%). Figure 3C, demonstrates the increase in the percent of cytochrome c release from 32.1% in control cells to 57.18% in doxorubicin and to 47.29% in tunicamycin treated cells ( *p* < 0.006). We analyzed CHOP/GADD153 expression involved in UPR regulated apoptosis. Figure 3D demonstrates significant increase in expression of CHOP/GADD153 from 22.27 ± 2.93% in non treated cells to 92.04 ± 1.2% in doxorubicin and 47.64 ± 2.44% in tunicamycin treated cells ( *p* < 0.001).

### Blocking of the cell surface GRP78 receptor inhibits cell apoptosis

**Figure 4:**
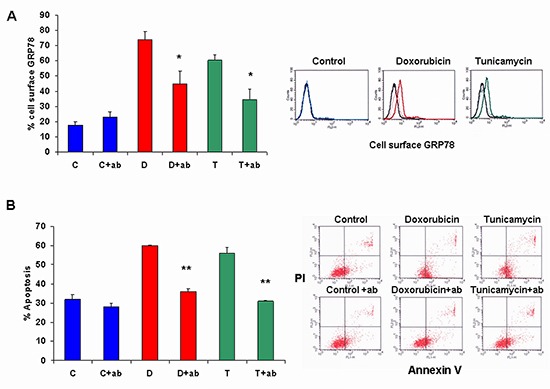
Blocking of cell surface GRP78 by anti-GRP78 antibody diminished doxorubicin and tunicamycin induction of apoptosis **(A)** Cell surface GRP78 in doxorubicin (d) and tunicamycin (t) treated cells was inhibited significantly by blocking of cell surface GRP78 by anti-GRP78 antibody (+ab) (*p < 0.01). No changes were observed in control cells (c). **(B)** Apoptosis was assessed by FACS analysis using Annexin/PI. Blocking of cell surface GRP78 (+ab) diminished apoptosis in doxorubicin (d) and tunicamycin (t) treated cells (**p < 0.008). In the upper right flank, representative FACS analysis of cell surface GRP78. Black line: Isotype, filled colored line: drug treated cells, dashed line: cell surface GRP78 blocked by anti GRP78 antibody.

In order to correlate the increase in cell surface GRP78 to apoptosis, we blocked the GRP78 receptor on 4T1 cells induced by the drugs using anti GRP78 antibody followed by the assessment of apoptosis. Figure 4A demonstrates that binding of anti GRP78 antibody blocked over 55.0% the GRP78 cell surface receptor on 4T1 cells ( *p* < 0.01).

Figure 4B demonstrates that blocking with anti-GRP78 antibody significantly inhibited cell apoptosis in doxorubicin and tunicamycin treated cells ( *p* < 0.008). This antibody was tested in various concentrations on 4T1 cells for cell proliferation. Results showed no change in cell proliferation (2.006 ± 0.001 in control cells vs 2.105 ± 0.048 with 0.01 μg/ml, 2.088 ± 0.102 with 0.1 μg/ml, 2.062 ± 0.062 with 1 μg/ml, 2.118 ± 0.040 with 10 μg/ml and 2.162 ± 0.010 with 20 μg/ml antibody).

### Anti GRP78 antibodies in the serum of mice

**Figure 5:**
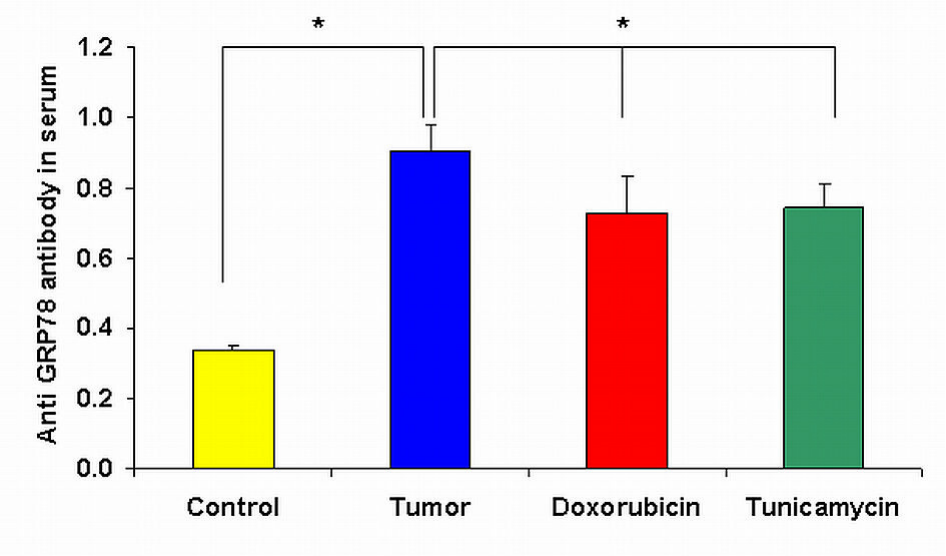
Anti GRP78 antibodies in the serum of mice Analysis of anti-GRP78 IgG antibodies titer in serum of mice by ELISA on day 31 after 4T1 inoculation shows a significant decrease in antibody titer of mice injected with 4T1 cells treated either with doxorubicin or tunicamycin in comparison to tumor control mice (*p < 0.001).

In order to find out whether the inoculation of cell surface GRP78 positive tumor cells induce anti-GRP78 antibodies in mice sera, we analyzed the serum of mice for anti-GRP78 IgG antibodies. Figure 5 demonstrates a significant increase in the antibody titer from 0.3 ± 0.014 in mice without tumors to 0.91 ± 0.07 in mice injected with non-treated 4T1 cells. Mice injected with 4T1 cells pre-treated either with doxorubicin or tunicamycin showed a significant decrease in anti-GRP78 antibody titers compared to mice injected with non-treated 4T1 cells ( *p* < 0.001).

## DISCUSSION

Cell surface GRP78 was identified on receptors positive BT474 breast cancer cell line but not on the triple negative MDAMB468 cells. We demonstrated that GRP78 negative expression on breast cancer tumor cells correlated with the absence of ER, PR and HER2 receptors in this TNBC. Although we focused our study to the MDAMB468 TNBC line, others also described low levels of GRP78 expression in TNBC cells lines such as MDAMB231 [29], HCC1937 and BT-20 [30,31]. We first evaluated the effect of doxorubicin and taxotere on cell surface GRP78 expression in the human breast cancer cell line, since we have previously shown that these drugs induced cell surface GRP78 expression in human colon cancer cells [32]. In this study we demonstrated a significant increase in cell surface GRP78 on the TNBC MDAMB468 by doxorubicin and taxotere but not on the receptor positive BT474 cells which probably reached the maximum expression before treatment. Treatment of the positive and negative cell lines with the different drugs revealed a diverse response to cell survival. BT474 was more sensitive to taxotere than MDAMB468, whereas doxorubicin's effect was similar in both cell lines. Knowing that the levels of the three receptors in the basal-like tumors are absent and that in general, these tumors exhibit a metastatic pattern, a poor prognosis and few treatment options compared to other subtypes [33,34], we proceeded to study the significance of cell surface GRP78 expression in the mouse 4T1 breast cancer cell line, a model used extensively for basal-like breast cancer [35]. We used the mouse 4T1 breast cancer model using Balb/C mice with a complete immune system in preference to human cell lines that require mouse deficient in CD3 (nude) for *in vivo* human cancer cell experiments [36]. We demonstrated that the 4T1 cell line displayed approximately 5% of cells expressing cell surface GRP78 which was significantly increased by doxorubicin, at similar levels to that of MDAMB468. To reveal the role of cell surface GRP78 expression in apoptosis, we compared the effect of doxorubicin to tunicamycin, two drugs involved in UPR activation by different mechanisms. Doxorubicin intercalates DNA and stops cell replication by disrupting the UPR at the level of activating transcription factor 4 (mediator of the UPR) and CHOP [37,38]. Our results were supported by other studies that suggest increased CHOP/GADD153 activity in response to doxorubicin treatment [39, 40].

Tunicamycin induces UPR by blocking the synthesis of all N-Glycans and causes cell apoptosis by increasing CHOP/GADD153 and GRP78 [41, 42].

In the tumor microenvironment, physiological stress such as lack of glucose and oxygen have been shown to increase GRP78 expression in order to facilitate cell survival by blockage of pro-apoptotic or activation of pro-survival pathways [12, 20–26]. A recent study suggests that metabolism deficiency promoting increase in GRP78 is related to stress induced apoptosis [43]. In our previous study on two colon cancer cell lines, cell surface GRP78 was not induced by metabolic deprivation [32].

In contrast to metabolic deprivation, both doxorubicin and tunicamycin induced over-expression of cell surface GRP78 causing a significant increase in stress induced apoptosis in TNBC cell lines. In a recent study in breast cancer, CHOP/GADD153 over-expression correlates with a significantly lower risk of recurrence in the GRP78-positive subset [44]. Additionally, a number of studies suggested that the expression of GRP78 in tumor samples may correlate with an improved prognosis such as neuroblastic tumors and lung cancer [45, 46]. It was demonstrated that the total expression of GRP78 is not altered by doxorubicin [40]. However, it was found that doxorubicin induced the synthesis of a GRP78 isoform that becomes inactive and cannot bind to misfolded proteins [40]. Even though the mechanisms responsible for the translocation of this protein, from the endoplasmic reticulum to the cell surface, remain poorly understood, it is possible that the inactive isoform of GRP78 is linked to the cell surface GRP78 expression that is associated to the induction of the pro-apoptotic factor CHOP/GADD153.

To further analyze our results obtained *in vitro*, doxorubicin and tunicamycin were used to explore their effect on tumor growth in mice. The treated tumor cells inoculated into mice resulted in inhibited tumor growth. These results are in accordance to our previous study on colon cancer that demonstrated inhibition of tumor growth and tumor metastases in mice transplanted with magnetic beads separated GRP78 positive cells [32].

Doxorubicin at a low dose of 0.1 μg/ml significantly augmented cell apoptosis in 4T1 cells. At 1 μg/ml, the percent of cell necrosis was enhanced significantly, showing a toxic effect independent from the cellular stress pathway. Tunicamycin failed to induce apoptosis at a low dose (1 μg/ml). However, a high dose (10 μg/ml) achieved significant apoptosis (with a small percent of necrotic cells). Doxorubicin at low doses was described as an inducer of endoplasmic reticulum stress-induced apoptosis in the 4T1 cell line through BAX and caspase-7 activation [47]. The effect of tunicamycin’ on apoptosis was also corroborated in other reports where low doses induced proliferation and migration and higher doses induced apoptosis of melanoma tumor cell lines [48]. Furthermore, we extracted the cells from the tumor nodules of each group 31 days after tumor inoculation. Cell surface GRP78, caspase 3 activity and cytochrome c release, involved in the apoptosis pathway were determined [49–51]. Our results showed that tumor cells that have been drug treated expressed a significantly higher percent of cell surface GRP78. The elevated expression corresponded to a significantly reduced tumor growth. The increase in caspase 3 activity and cytochrome c release in drug treated tumor cells corroborates with the activation of apoptotic pathways involved in cellular stress [51–53]. Moreover, we also demonstrated that the endoplasmic reticulum apoptotic signaling was activated by tunicamycin and doxorubicin as evidenced by CHOP/GADD153 and cell surface GRP78 up-regulation [53].

Other compounds proposed for cancer therapy induced up regulation of GRP78 and GADD153 [54].

The blocking of cell surface GRP78 by anti-GRP78 antibody prevented apoptosis, suggesting that induction of cell surface GRP78 by doxorubicin and tunicamycin is required for apoptosis. It was described that anti GRP78 antibodies binding the N-terminal promote cell proliferation, while antibodies binding the C-terminal promote cell apoptosis [55]. The antibody used for blocking in this study was a commercial antibody produced using a synthetic peptide corresponding to near N-terminus of human GRP78. This antibody in various concentrations did not induce MDAMB468 and 4T1 cell proliferation.

It is important to note that TNBC treatment consists of high dose chemotherapy that was shown to have a toxic effect on other tissues and undesirable serious cardio toxic side effects [40,56]. Thus, cell surface GRP78 modulation by low concentrations of chemotherapy might be valuable in the treatment of TNBC for which at present are lacking therapeutic opportunities. The significance of cell surface GRP78 expression in breast cancer cells in this study was also evaluated at the level of anti GRP78 antibody production in the serum of mice inoculated with 4T1 cells. It is known that the expression of cell surface GRP78 induces a humoral response. Anti-GRP78 auto-antibodies have recently been identified as biomarkers and correlated positively with cancer progression in prostate, ovarian and colon cancers [57–60]. In our study we showed a significant increase of anti GRP78 auto-antibodies in the serum of mice injected with 4T1 compared to mice without tumor. The presence of anti GRP78 antibodies in the serum of tumor-bearing mice is due to the significant rise in cell surface GRP78 from 5 to 20 percent in 4T1 cells extracted from mice tumor nodules. Treatment of cells with doxorubicin or tunicamycin significantly decreased the titer of anti GRP78 antibodies. However, the titer was significantly higher than in mice without tumors. Our results concur with a study performed in a mouse model of Lewis lung carcinoma cells treated with cyclophosphamide that inhibited tumor growth and induced a reduction in the serum titer of anti-GRP78 which also decreased with time [61]. In the present study significant higher cell surface GRP78 in doxorubicin and tunicamycin treated cells correlated with a significantly lower titer of anti GRP78 antibodies in serum compared with non-treated tumor bearing mice.

In conclusion, we demonstrate the significance of the association of increased cell surface GRP78 with tumor growth inhibition, tumor cell apoptosis and reduced anti GRP78 antibodies. A better understanding of stress induction of apoptotic signaling in triple negative breast cancer cells may help to define new therapeutic strategies.

## METHODS

### Ethics Statement

Investigation has been conducted in accordance with the ethical standards and according to the Declaration of Helsinki and according to national and international guidelines and has been approved by the authors' institutional review board at Tel Aviv University, Tel Aviv, Israel.

### Cell lines

4T1 is a mouse breast cancer tumor cell line from mammary gland tissue that very closely mimics human breast cancer; MDAMB468 is a human breast cancer cell line, both known as triple negative: Her2, ER and PR negative. BT474 is a human breast cancer cell line ER, PR and HER2 positive. Cells were grown in DMEM supplement with 10% FCS, L-glutamine (2 mM), Na-pyruvate (1 nMol), penicillin (100 units/ml), streptomycin sulfate (0.1 mg/ml) and nystatine (12.5 μ/ml) (complete culture medium) (Biological Industries, Kibbutz Beit HaEmek, Israel). Cultures were maintained at 37°C in a humidified 5% CO2 incubator. A large stock of cells was prepared to maintain the homogeneity and tumorigenicity of the cell lines.

### Drugs

Doxorubicin (0.01, 0.1, 1 and 10 μg/ml) and taxotere (1, 5, 10 and 50 μg/ml) were added to cultures. Tunicamycin (1 and 10 μg/ml) was also inserted to the culture. All drugs were combined with cultured cells for 48 hs. The various concentrations of the drugs were determined in preliminary assays.

### Determination of cell surface GRP78 and apoptosis by fluorescence-activated cell sorting (FACS)

Tumor cells (200,000 cells/dish) were incubated for 48 hs with or without drugs in 60 mm Petri dishes and grown in complete culture medium for additional 24 h in duplicates. Cells were detached by cold PBS and stained with 2 μg/ml polyclonal rabbit anti GRP78 antibody (Santa Cruz Biotechnologies, CA, USA) for 1 h at 4°C, followed by anti-rabbit fluorescein antibody (Jackson Immunoresearch, West Grove, PA, USA) for an additional 30 min. Isotype control was determined by FITC conjugated anti-rabbit to determine nonspecific binding. Gated live cells were analyzed using FACS (Beckton Dickinson, Franklin Lakes, NJ, USA) and Cell Quest software (Beckton Dickinson). For cell surface GRP78 blocking, rabbit anti GRP78 (Thermo Scientific, PA1-37805, IL, USA) was added at 1 μg/ml overnight and cells were stained for FACS analysis using goat anti GRP78 followed by anti-goat phycoerythrin (PE) with minimal cross reaction to rabbit. The mean percent of cell surface GRP78 positive stained cells from 6 experiments was determined ± SE. For apoptosis, cells were harvested and stained with Annexin-V-FITC (Mebcyto Apoptosis kit, MBL, Naka-Ku Nagoya, Japan) following the manufacturer's instructions. The AnnexinV/PI stained cells were scored as apoptotic cells. Isotype control was determined by nonspecific binding of unstained cells. Results are presented as the mean percent of apoptotic cells ± SE in 4–6 separate experiments.

### Tumor cell proliferation

Proliferation of cells was analyzed by XTT cell proliferation kit (Biological Industries, Kibbutz Beit HaEmek, Israel). Tumor cells (5000 per well) were incubated in 96 well plates in triplicates in 100 μl complete culture medium for 24 hours for cell adhesion. Thereafter, the culture medium was removed and cells were exposed to different concentrations of the drug compounds at the concentrations described above or anti GRP78 antibody at 0.01, 0.1, 1, 2, 10 and 20 μg/ml during 48 hs. XTT reaction solution was added to the cultures for 5 hs in order to measure absorbance at 450–500 nm in an ELISA reader. The results of 6 repeated experiments are presented and scaled as optical density unit ± SD.

### Colony formation

Tumor cells were seeded (1000 cells/well) in triplicates onto 6 well plates for 24 hs in complete culture medium. Drugs were added as described above. Media was replaced every other day, and after 2 weeks, colonies were stained using gentian violet. Pictures were taken and results were calculated using Image-Pro Plus software (Media Cybernetics, Silver Spring, MD, USA). Results of 4 experiments are presented as numbers of colonies ± SD.

### Murine tumor sub-cutane nodules model

The experiments were performed according to the Institutional Animal Care and Use Committee of Tel Aviv University conforming to the NIH Guide for the Care and Use of Laboratory Animals.

Thirty 10-week old Balb/C mice were inoculated subcutaneously in the right flank with 1 × 10^6^ 4T1 cells/100 μl PBS. The cells were previously incubated for 48 hs with either doxorubicin at 0.1 μg/ml (group 2, n = 10) or tunicamycin at 10 μg/ml (group 3, n = 10). Cells without treatment were considered as control (group 1, n = 10). Caliper measurements of the longest perpendicular tumor diameters were performed every alternate day to estimate the tumor volume using the following formula: 4/3 × (width/2)2 × (length/2). Tumor growth was evaluated from when palpable until day 31 after tumor inoculation. Results are presented in cm^3^ ± SE.

### FACS analysis for caspase 3 activity, cytochrome c release and CHOP/GADD153 expression

Tumor nodules were extracted from 5 mice in each group 31 days after tumor inoculation. The tumors were placed into 5ml PBS containing antibiotics. The skin was removed and the portion was sliced into fine pieces with scalpel blades and transferred into 50 ml tubes. After centrifugation 1500 rpm for 10 min, 0.25% trypsin was added and placed for 5 minutes on rocker. Cells obtained after centrifugation were suspended in PBS containing 5% FCS and 0.1% Na-azide. Cells were counted and transferred to FACS tubes for the evaluation of cell surface GRP78, caspase 3 activity, cytochrome c release and CHP/GADD153 expression.

To determine caspase-3 activity we used CaspGLOW™ Fluorescein Active Caspase-3 Staining Kit (e-Bioscience, CA, USA) following manufacturer's instructions. Cytochrome c release was detected by FlowCellect™ Cytochrome *c* Kit (Millipore, CA, USA) following manufacturer's instructions.

The FACS analysis kit used in this study for cytochrome c release determines the percent of the viable cells with higher levels of cytochrome *c* staining. Apoptotic cells which have released their Cytochrome *c* to the cytoplasm will demonstrate reduced staining intensity when probed with the anti-cytochrome *c-* FITC antibody.

To determine CHOP/GADD153 expression, cells were fixed with 4% paraformaldehide for 10 minutes, washed and permeabilized (by Permeabilization/wash buffer (R&D Systems, MN, USA) following manufacturer's instructions. Then, cells were exposed to monoclonal anti GADD153 antibody (Santa Cruz Biotechnologies, CA, USA) followed by anti mouse FITC. Previous to the fixation, cells were blocked with a monovalent Fab fragment of anti-mouse IgG.

### Determination of anti GRP78 antibody in mouse serum by ELISA

Five mice from each group were bled before sacrifice and serum was collected and frozen (−80ºC). ELISA 96-well high binding plates were coated with 5 μg/ml recombinant GRP78 diluted in 0.1 mol/L Na_2_CO_3_, 0.01%NaN_3_ (pH 9.3) a day before assay. Plates were blocked with PBS-10% BSA for 1 h at room temperature. Serum, defrosted immediately before assay, was diluted in PBS at 1:10, 1:100, and 1:1000. Fifty μl/well of the diluted serum was placed in triplicates in the blocked coated plates for 2 hs at 37ºC followed by 2 washes with PBS. A secondary anti human IgG biotinylated antibody (BethylLaboratories, Montgomery, TX, USA) was added at 1 μg/well for 2 hours at room temperature. After intensive washes, streptavidin HRP (R&D Systems, Minneapolis, MN, USA) was added to plates for 1h at room temperature. One step TMB substrate system (eBioscience, San Diego, CA, USA) was added at 100 μl/well in dark until color developed. To stop the reaction, 50 μl/well HCl 1M was inserted into each well. The reading method: 450 nm in an ELISA reader and the results (from dilution 1:100) are presented in arbitrary numbers ± SE.

### Statistical analysis

One way Anova and Tukey tests were used to assess the association between the groups and different variables. A p value of <0.05 was considered statistically significant.
